# The Impact of Western Diet and Nutrients on the Microbiota and Immune Response at Mucosal Interfaces

**DOI:** 10.3389/fimmu.2017.00838

**Published:** 2017-07-28

**Authors:** Donjete Statovci, Mònica Aguilera, John MacSharry, Silvia Melgar

**Affiliations:** ^1^APC Microbiome Institute, University College Cork, Cork, Ireland; ^2^School of Microbiology, University College Cork, Cork, Ireland

**Keywords:** westernized diet, inflammatory bowel disease, asthma, saturated fat, micronutrients, microbiota

## Abstract

Recent findings point toward diet having a major impact on human health. Diets can either affect the gut microbiota resulting in alterations in the host’s physiological responses or by directly targeting the host response. The microbial community in the mammalian gut is a complex and dynamic system crucial for the development and maturation of both systemic and mucosal immune responses. Therefore, the complex interaction between available nutrients, the microbiota, and the immune system are central regulators in maintaining homeostasis and fighting against invading pathogens at mucosal sites. Westernized diet, defined as high dietary intake of saturated fats and sucrose and low intake of fiber, represent a growing health risk contributing to the increased occurrence of metabolic diseases, e.g., diabetes and obesity in countries adapting a westernized lifestyle. Inflammatory bowel diseases (IBD) and asthma are chronic mucosal inflammatory conditions of unknown etiology with increasing prevalence worldwide. These conditions have a multifactorial etiology including genetic factors, environmental factors, and dysregulated immune responses. Their increased prevalence cannot solely be attributed to genetic considerations implying that other factors such as diet can be a major contributor. Recent reports indicate that the gut microbiota and modifications thereof, due to a consumption of a diet high in saturated fats and low in fibers, can trigger factors regulating the development and/or progression of both conditions. While asthma is a disease of the airways, increasing evidence indicates a link between the gut and airways in disease development. Herein, we provide a comprehensive review on the impact of westernized diet and associated nutrients on immune cell responses and the microbiota and how these can influence the pathology of IBD and asthma.

## General Introduction

The prevalence of chronic inflammatory diseases affecting mucosal sites such as the intestine and the airways is increasing worldwide (1, 2). Among these, inflammatory bowel disease [IBD, mainly comprising ulcerative colitis (UC) and Crohn’s Disease (CD)] and allergic asthma are the most relevant. Recent findings point toward potential links between these two pathologies, e.g., histamine and mast cell activity and immunoglobulin E (IgE) production, reviewed in Ref. (3). Both diseases have a multifactorial cause, in which environmental factors such as diet and the commensal microbiota are gaining increased attention. In this regard, consumption of the so-called “Westernized” diet is associated with increased risk for IBD (4) and asthma morbidity (5). Westernized diet is characterized by a high content of proteins (derived from fatty domesticated and processed meats), saturated fats, refined grains, sugar, alcohol, salt, and corn-derived fructose syrup, with an associated reduced consumption of fruits and vegetables (4, 6, 7). Research in the last decade has uncovered that changes from a diet rich in fibers and low in fats to a diet low in fibers and high in saturated fats directly contributes to the development of obesity, metabolic syndrome, and cardiovascular diseases (8, 9). Macronutrients (carbohydrates, lipids, and protein) and micronutrients (vitamins and minerals) are required for our body to function and several of these are naturally obtained from our diets and from the resident microbiota. Both patients with IBD and asthma present nutritional problems leading to several complications including anemia, osteoporosis, acute respiratory infections, etc. The link between diet, nutrients and immune responses is embedded in a complex network of signals and has to be considered in the light of other factors including microbial composition, genetic background, and lifestyle, to mention but a few. The advent of high-throughput Next-Generation Sequencing technologies has driven the discovery and dissection of regulatory mechanisms involved in the disease state. In this review, we will focus on the interactions between diets and nutrients associated with Westernized regimes and their impact on the microbiota and immune responses at mucosal interfaces, i.e., the intestines and lungs. We will outline the complex network between nutrients, microbial alterations and abnormal immune responses associated with IBD and asthma.

**Figure 1:**
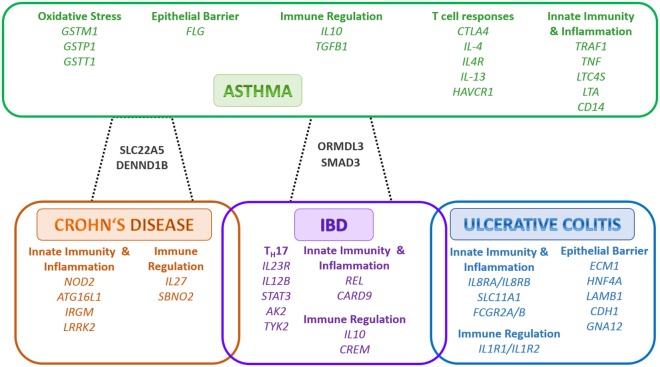
Shared and individual inflammatory bowel disease (IBD) and Asthma susceptibility genes/loci. The outlined genes are grouped according to function. In green color are the genes associated to asthma, in blue color are the genes associated to ulcerative colitis only, in orange color are the genes associated to Crohn’s disease only, in purple color are the genes associated to IBD, in black color are the genes associated to asthma and Crohn’s disease or asthma and IBD, respectively. Adapted from Ref. (37–40). Abbreviations: AK2, adenylate kinase 2; ATG16L1, autophagy related 16 like 1; CARD9, caspase recruitment domain family member 9; CD14, cluster of differentiation 14; CDH1, cadherin 1; CREM, CAMP responsive element modulator; CTLA4, cytotoxic T-lymphocyte associated protein 4; DENND1B, DENN domain containing 1B; ECM1, extracellular matrix protein 1; FCGR2A, Fc fragment of IgG receptor IIa; FCGR2B, Fc fragment of IgG receptor IIb; FLG, filaggrin; GNA12, G-protein subunit alpha 12; GSTM1, glutathione S-transferase mu 1; GSTP1, glutathione S-transferase pi 1; GSTT1, glutathione S-transferase theta 1; HAVCR1, hepatitis A virus cellular receptor 1; HNF4A, hepatocyte nuclear factor 4 alpha; IL-13, interleukin 13; IL-4, interleukin 4; IL-10, interleukin 10; IL-12B, interleukin 12B; IL-1R1, interleukin 1 receptor type 1; IL-1R2, interleukin 1 receptor type 2; IL23R, interleukin 23 receptor; IL-27, interleukin 27; IL-4R, interleukin 4 receptor; CXCR1, C–X–C motif chemokine receptor 1; CXCR2, C–X–C motif chemokine receptor 2; IRGM, immunity related GTPase M; LAMB1, laminin subunit beta 1; LRRK2, leucine rich repeat kinase 2; LTA, lymphotoxin alpha; LTC4S, leukotriene C4 synthase; NOD2, nucleotide binding oligomerization domain containing 2; ORMDL3, ORMDL sphingolipid biosynthesis regulator 3; REL, REL proto-oncogene, NF-κB subunit; SBNO2, strawberry notch homolog 2; SLC11A1, solute carrier family 11 member 1; SLC22A5, solute carrier family 22 member 5; SMAD3, SMAD Family Member 3; STAT3, signal transducer and activator of transcription 3; Th, T helper cell; TGFB1, transforming growth factor beta 1; TNF, tumor necrosis factor; TRAF1, TNF receptor associated factor 1; TYK2, tyrosine kinase 2.

**Figure 2:**
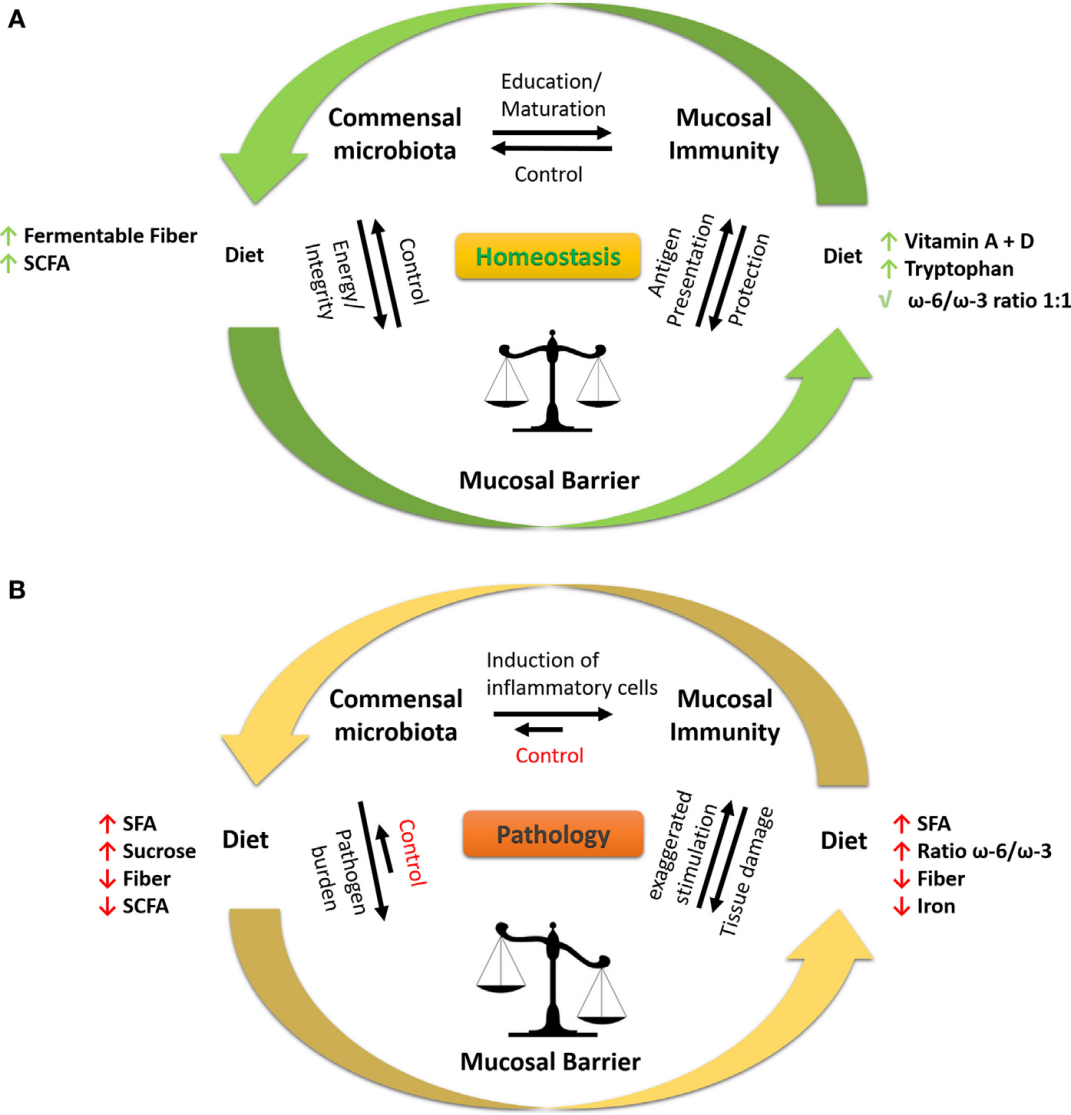
Interaction between diet, microbiota, and immune response at mucosal sites. **(A)** To keep a healthy state, the local microbiota and mucosal immune system are in homeostasis at mucosal sites. The microbiota educates and promotes the maturation of the immune system by induction of pro-inflammatory and anti-inflammatory immune cells, e.g., Th17 (SFB), T regulatory cells (*Clostridia* spp.), and Th1 (*Bacteroides fragilis*). Moreover, the immune system surveys microbial activities (e.g., antigen sampling at the mucosal barrier) and responds in a controlled fashion by producing, e.g., antimicrobial peptides, sIgA to prevent tissue damage. The integrity of the mucosal barrier is sustained by bacteria-produced metabolites (e.g., SCFA) such as butyrate resulting in high expression of tight-junction proteins and mucus production, thereby restricting interaction of microbes to the lumen and luminal epitheliums. The diet is involved in all processes, serving the microbiome with fermentable fibers and the immune system and epithelium with essential nutrients, e.g., vitamins and minerals. **(B)** During pathological conditions, such as inflammatory bowel disease and asthma, the homeostasis at the mucosal barrier is disrupted. A westernized diet, i.e., high in SFA, high ω-6/ω-3 ratio, high sucrose and iron (oral iron supplements), and low in fiber promotes inflammation and growth of pathogenic/pathobiont (disease causing) bacteria in the gut. The microbiota, which is rich in non-beneficial bacteria, favorably induces the maturation of pro-inflammatory immune cells, leading to uncontrolled inflammation resulting in tissue damage of the mucosal compartment. The damaged mucosa and shifted immune response fail to control the microbiota, which exaggerates the pathophysiological state. Under certain conditions, bacteria-derived LPS enters the systemic circulation and further stimulates the immune system toward a pro-inflammatory state. Abbreviations: LPS, lipopolysaccharide; SCFAs, short-chain fatty acids; SFAs, saturated fatty acids; SFB, segmented filamentous bacteria; sIgA, secretory immunoglobulin A; ω-6/ω-3, omega-6/omega-3 fatty acid ratio; Th, T helper.

**Figure 3:**
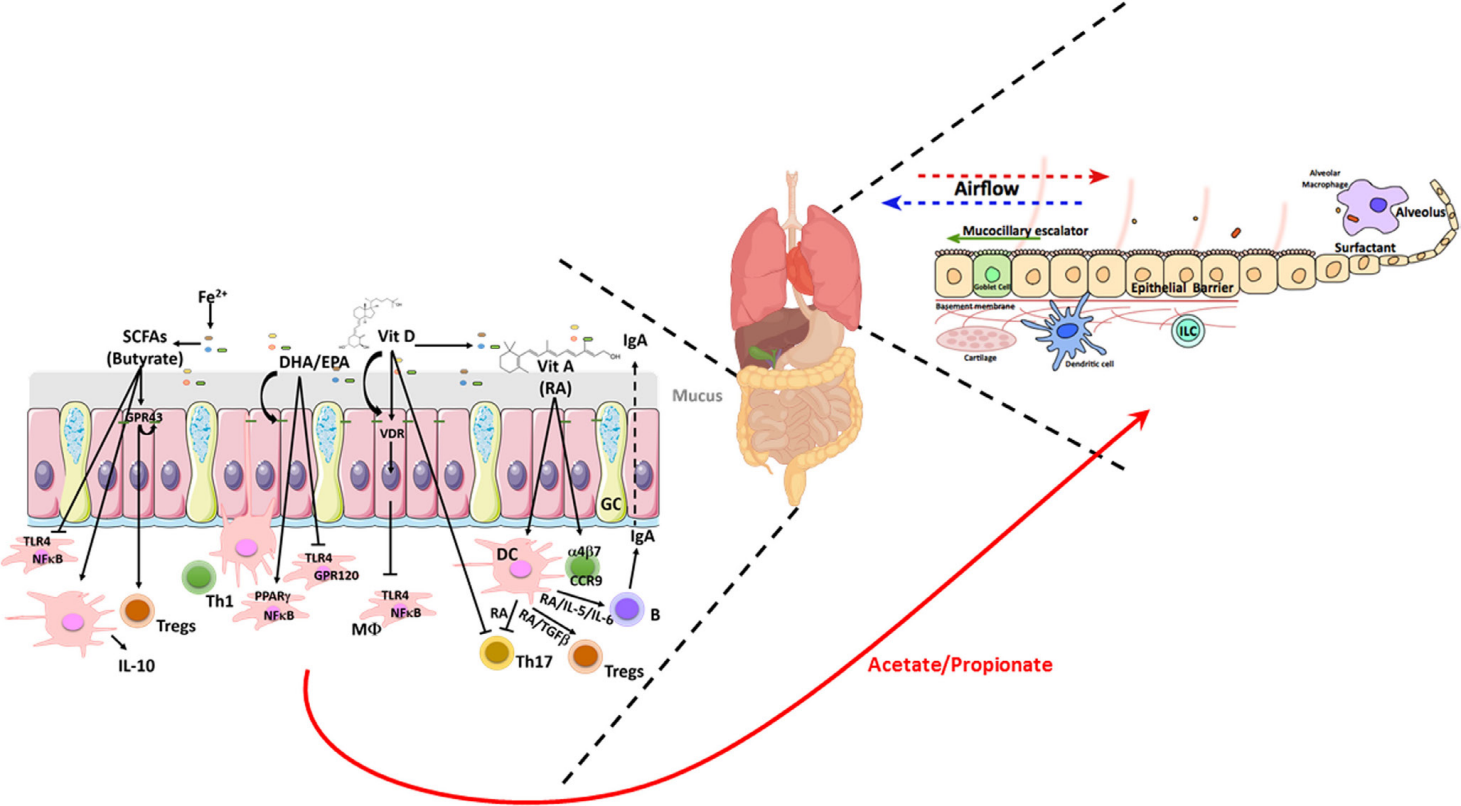
Schematic illustrating the nutrient factors regulating microbial and host responses in the healthy gut and lung. Homeostatic balance at the mucosa due to a balanced diet rich in fiber allows for regulated interactions between the epithelia and the microbiome. This dialog with the microbiome allows for appropriate epithelial barrier function, mucus secretion, and underlying immune sensing. In the gut, a balanced microbiome generates SCFAs and dietary long chain FAs and the fat-soluble vitamins A and D which induce a tolerogenic mucosal immune state locally at the gut but also systemically and particularly in the lung. The gut-derived SCFAs acetate and propionate enhance DCs, ILC, and macrophage phagocytic function and Tregs balance resulting in the control of lung microbiota and efficient mucocillary clearance of inhaled microbes and particulates. Lung figure adapted from Ref. (41). Abbreviations: CCR9, C–C motif chemokine receptor 9; DCs, dendritic cells; DHA, docosahexaenoic acid; EPA, eicosapentaenoic acid; FAs, fatty acids; GC, goblet cells; GPR, G-protein coupled receptor; ILC, innate lymphoid cells; α4β7, integrin α4β7; IL-1β, interleukin 1 beta; IL-4, interleukin 4; IL-5, interleukin 5; IL-10, interleukin 10; Fe2^+^, iron; MΦ, macrophage; NFκB, nuclear factor kappa-light-chain-enhancer of activated B cells; PPARγ, peroxisome proliferator-activated receptor gamma; RA, retinoic acid; SCFAs, short-chain fatty acids; TGFβ, transforming growth factor beta; Th, T helper; Tregs, T regulatory cells; TLR4, toll-like receptor 4; VitA, vitamin A; VitD, vitamin D; VDR, vitamin D receptor; healthy bacteria phyla—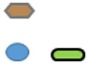, bacteroides; 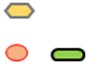, firmicutes; 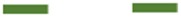, barrier integrity.

**Figure 4:**
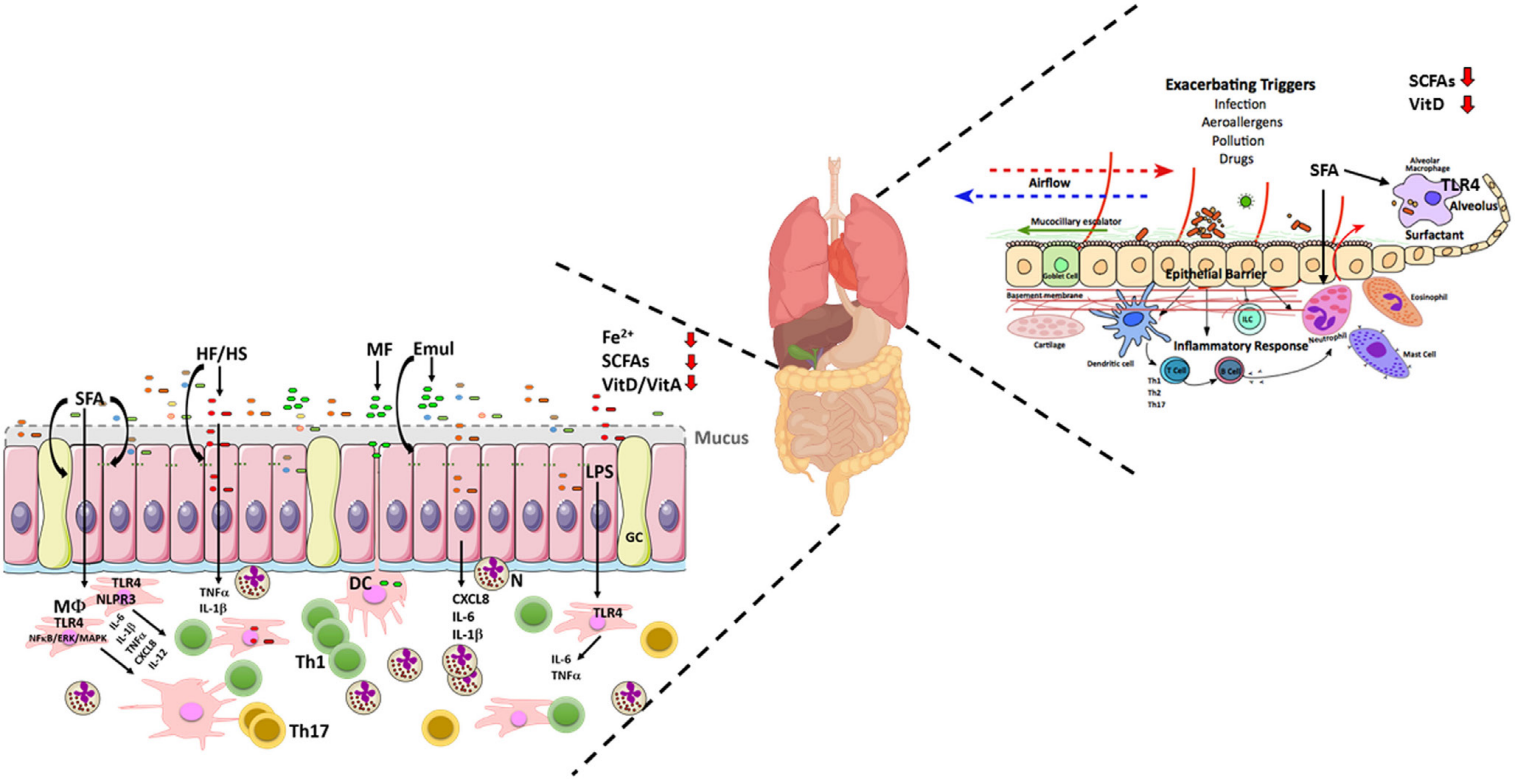
Schematic illustrating the nutrient factors affecting microbial and host responses in the inflamed gut in inflammatory bowel disease (IBD) and the lung in Asthma. In both IBD and Asthma genetic susceptibility, microbiota, and dietary changes result in disease development and inflammation. Dysfunctional epithelia barrier function allows for malabsorption of nutrients, inappropriate immune sampling, and colonization of the gut by pathobionts and subsequent disease exacerbation. In the lung, environmental triggers stimulate inflammatory and allergic reactions resulting in mucus hypersecretion, epithelia, and tissue remodeling and resulting compromised of lung function. This microenvironment change allows for microbial changes which allow for increased respiratory infections in asthmatic patients. Both IBD and asthma pathogenesis is related to reduced microbiota-derived SCFAs, malabsorption of iron and Vitamins and reduced gut-derived SCFA result in a trend toward an inflammatory sensing of the mucosa associated microbiota. A diet high in SFA increases TLR4 sensing and subsequent inflammatory reactions to the microbiota resulting in disease progression. This dysregulated mucosal inflammation changes the epithelia barrier function and subsequently alters the microbiota of both sensitive immune sites displaying the characteristic phenotypes associated with both IBD and asthma. Lung figure adapted from Ref. (41). Abbreviations: CXCL8, C–X–C Motif Chemokine Ligand 8; DCs, dendritic cells; Emul, emulsifier; GC, goblet cell; HF/HS, high-fat/high sucrose; ILC, innate lymphoid cells; IL-1β, interleukin 1 beta; IL-6, interleukin 6; IL-12, interleukin 12; Fe2^+^, iron; LPS, lipopolysaccharide; MΦ, macrophage; MAPK/ERK, mitogen-activated protein kinase/extracellular signal-regulated kinase; MF, milk fat diet; N, neutrophils; NFκB, nuclear factor kappa-light-chain-enhancer of activated B cells; PPARγ, peroxisome proliferator-activated receptor gamma; SFA, saturated fatty acids; SCFAs, short-chain fatty acids; TGFβ, transforming growth factor beta; Th, T helper; TNF, tumor necrosis factor; TLR4, toll-like receptor 4; VitA, vitamin A; VitD, vitamin D; healthy bacteria phyla— 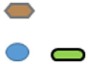, bacteroides; 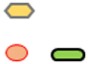, firmicutes; 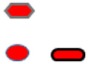, adherent and invasive *Escherichia coli* (AIEC); 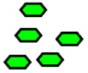, *Bilophila wadsworthia*; 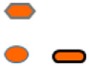, other altered bacterial spp; 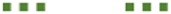, compromised barrier integrity.

Table 1 summarizes the impact of dietary factors on host responses. Figure 1 summarizes identified genes associated with IBD and asthma and immune responses. Figure 2 displays the regulatory interaction between diet, mucosal immunity, and commensal microbiota to maintain mucosal homeostasis and the resulting pathology upon loss of balance. Figures 3 and 4 outline the mechanisms targeted by nutrients in the healthy intestine and lung and in the inflamed gut (IBD) and lung (asthma), respectively.

**Table 1:** Dietary factors and host inflammatory responses at mucosal sites.

Dietary factor	Inflammatory/immune response		Reference
	Intestine	Lung	
**Macronutrients**			
**Fat**
High-fat diet	Permeability of epithelial barrier ↑	Neutrophil ↑ TLR4 mRNA ↑	(10, 11)

Saturated fatty acids	Activation of TLR4↑		(12)

n-3 PUFA, e.g., EPA, DHA	PG (series-3) ↑ LK (series-5) ↑ Leukocyte chemotaxis↓ IL-1β ↓, TNFα ↓ Resolvins, Maresins and Protectins↑	Maternal supplementation reduced Childhood Asthma IL-13 cord blood ↓ Mucus (murine) ↓ CD45^+^ inflammatory cell infiltrates (murine) ↓	(13) (14, 15)

n-6 PUFA, e.g., ARA, linoleic acid	PG (series-2) ↑ LK (series-4) ↑	Mucus (murine) ↓	(14)

SCFA, e.g., butyrate	Energy source for colonocytes Barrier function ↑ Peroxisome proliferator-activated receptor γ activation ↑	Allergy ↓	(16, 17)

**Carbohydrates**
Sucrose	Permeability of epithelial barrier ↑	Asthma and dental caries ↑	(18–21)

Fermentable carbohydrates, e.g., fiber	Butyrate production ↑	SCFA levels ↑ DC maturation ↓ T_H_2 response ↓	(22, 23)

**Proteins**
Animal-derived proteins, e.g., carnitine	TMAO synthesis ↑	Asthma exacerbation (cured meats)	(24, 25)

Dipeptides, e.g., alanine–glutamine	Mucin 2 expression ↑		(26)

**Micronutrients**			

**Vitamins**			
Vitamin A, e.g., RA, β-carrotene	Induction of tolerogenic DC and Tregs ↑		(27, 28)

Vitamin D	Cathelicidin production Innate defense toward regulatory state ↑ Ca^2+^absorption ↑	Maternal supplementation reduction in airway smooth muscle	(27, 29, 30)

Vitamin B, e.g., thiamine, folate, cobalamine, pyridoxine	Vitamin B9 deficiency—colonic Foxp3 + Tregs ↓	Folate deficiency—asthma exacerbations	(31–34)

**Minerals**
Iron	Incorporated into iron–sulfur clusters, redox cofactors, or used metalloenzymes		(35, 36)

*ARA, arachidonic acid; Ca^2+^, calcium; DHA, docosahexaenoic acid; EPA, eicosapentaenoic acid; Foxp3, Forkhead-Box-Protein P3; LK, leukotrienes; PG, prostaglandins; RA, retinoic acid; TLR4, toll-like receptor 4; PUFA, polyunsaturated fatty acid; SCFA, short-chain fatty acid; Tregs, T regulatory cells; CD, cluster of differentiation; DC, dendritic cells; IL, interleukin; TMAO, trimethylamine-N-oxide; Th, T helper cell*.

## IBD—Genetics, Immune Response, and Microbiota

Inflammatory bowel diseases are multifactorial chronic immune-mediated diseases of the gastrointestinal (GI) tract. They often have an early onset and a course which is characterized by intermittent phases of remission and relapses (42, 43). Nearly 2.2 million people in Europe, 1.5 million Americans, and several 100,000 more individuals suffer from IBD. The prevalence and incidence of IBD is increasing worldwide and its growth is not confined to Western Europe or the USA; countries adapting a westernized lifestyle, such as Japan and South Africa, are seeing mounting numbers of affected individuals (2). Patients suffering from IBD present symptoms such as abdominal pain, fever, and diarrhea with blood and/or mucus excretion. Antibiotics, biologics, corticosteroids, and immune modulators are the current treatments of choice for patients with IBD, with the overall goal being inflammation reduction, increase of mucosal healing and symptom relief (16, 44). New treatment strategies include inhibitors targeting leukocyte trafficking (α4β7 integrin, sphingosine-1-phosphate, S1P), cytokine [interleukin (IL)-12p40, IL-23], janus kinase-pathway, inflammasome (NLRP3/IL-1β), to name a few (45, 46). The etiology of IBD is still unknown but the evidence points toward environmental factors, with the microbiota being of particular interest. The microbiota can trigger and/or sustain a tissue damaging immune response in genetically susceptible individuals. Research in the last decade has identified a crucial role for the commensal bacteria in the pathogenesis of IBD. Experimental IBD was not evidenced in animals raised under germ-free (GF) conditions when compared to groups of conventionalized animals (47). Antibiotics have proven beneficial to certain subgroups of patients with CD but not in patients with UC (48) and inflammatory lesions are more frequently located in areas with large bacterial burden, i.e., ileum and colon (49). Alterations in the enteric microbial flora reported in patients with IBD include decreases in *Firmicutes* and *Bacteroidetes* and increases in *Enterobacteriaceae* (e.g., *Escherichia coli*) (50). A reduction in anti-inflammatory commensals, such as *Faecalibacterium prausnitzii* has been associated with CD (51). Several pathogenic bacteria are suggested as etiological agents of IBD but to date none has been identified to cause IBD (47). In contrast, pathobionts, i.e., commensal bacteria with potential pathological properties, have been isolated, including strains of adherent and invasive *E. coli* (AIEC), commonly identified in the mucosa of CD patients (52).

Genome-wide association studies have so far identified over 160 genetic loci in IBD, with 30 loci being specific to CD, 23 loci to UC, and 110 loci are associated with both forms of IBD (53). IBD susceptibility single-nucleotide polymorphisms were identified in genes affecting innate and adaptive immune cell function, bacterial recognition, etc. (Figure 1). Therefore, the role of mononuclear phagocytes including monocytes/macrophages and dendritic cells (DCs) in the development of IBD has been extensively studied. Several mouse models of IBD, including dextran sodium sulfate (DSS)- and 2,4,6-trinitrobenzene sulfonic acid-induced colitis and the TNFΔARE model of Crohn’s-like ileitis, have revealed that lamina propria mononuclear phagocytes have protective as well as pathogenic roles during the disease progression (54–60). Three explanations have been postulated to explain these findings—(1) an inappropriate response to non-harmful commensal bacteria (i.e., NOD2, REL, CARD9); (2) an inefficient clearance of microbes (commensals/pathobionts) leading to chronic immune stimulation (i.e., ATG16L1, IRGM), and (3) a failure to resolve inflammation by maintaining a pro-inflammatory phenotype (i.e., IL-12, IL-18RAP/IL-1R1, IFNGR/IFNAR1) (39) (Figure 1). The intestinal epithelium functions as a barrier between the host and its environment (microbes, non-self-antigens from diet, nutrients, etc.) and consists of highly specialized cells that fulfill this barrier task. Genes associated with epithelial cell function, such as HNF4A, ECM1, CDH1 have a UC correlation (53) (Figure 1). Alterations in barrier integrity associated with IBD include decreased structure of tight-junction (TJ) proteins, which regulate paracellular permeability, impaired mucus production due to loss of goblet cells and an altered production of antimicrobial peptides (61) (Figure 4).

In terms of location, CD can affect any part of the GI tract from the mouth to rectum. However, in the majority of patients with CD the inflammation is localized to the distal ileum and proximal colon (62). The inflammation in CD is patchy and often transmural, which can lead to the development of fibrosis, fistulas, fissures, strictures, etc. A dense infiltration with macrophages and lymphocytes and granuloma formation is a typical feature of the disease. Patients with CD present an imbalanced immune response with high expression of innate pro-inflammatory cytokines, including IL-1β, IL-6, and tumor necrosis factor (TNF)-α and a T helper (Th)1 (IL-12-mediated interferon (IFN)γ) and Th17 (IL-17a) profile resulting in an enhanced and uncontrolled immune response (16, 43, 62) (Figure 4). In contrast to CD, UC is restricted to the mucosa of the colon and is associated with large infiltrates of neutrophils, T and B cells in the lamina propria. Characteristically, the inflammation originates in the rectum extending continuously in a proximal fashion. Crypt abscesses, formed by extravasation of neutrophils through the intestinal epithelium, ulcerations, and goblet cell loss are typical features of UC. Moreover, high levels of innate cytokines, including IL-1β, IL-6, and TNFα, and chemokines, such as CXCL8 and GROα/CXCL1 (neutrophil attractants), as well as an atypical Th2 cytokine profile accompanied by high production of IL-5, IL-10, transforming growth factor beta (TGFβ) and only initially IL-4 production, which is superseded by IL-13 production is associated to patients with UC (62–65) (Figure 4).

## Asthma—Genetics, Immune Response, and Microbiota

Asthma is an increasingly common heterogeneous chronic inflammatory disease, which places substantial burden on patients, their families, and the community (66). Asthma is characterized by airway immune hyper responsiveness to inhaled environmental particles leading to wheezing, breathlessness, chest tightness, and coughing effecting airway function (http://ginasthma.org/). Worldwide the incidence of asthma, is increasing, with an estimated 300 million affected individuals (http://ginasthma.org/). Once thought to be a childhood disease it is now presenting in respiratory clinics as first time adult onset asthma.

Asthma presents with airway inflammation following exposure to insults such as allergens, pollutants, and microbes (67). The primary site of immune induction is initially the lung epithelium, which interacts with the underlying antigen presenting cells such as DCs, inducing an immune response (Figure 4). Alveolar macrophages in the airway lumen act as clearance and immune sampling mechanisms at the interface between the mucosa and the external environment (68). The immune signaling from epithelial cells and macrophages results in secretion of first order cytokines, such as CXCL8, IFNα, IL-1β, IL-33, TGFβ, and thymic stromal lymphopoietin (TSLP), which induce a rapid immune trafficking and a clearance response which subsequently results in second order cytokine secretion by T cells (69). Activated DCs migrate to the lymph nodes and induce T cell activation (70) (Figure 4). The subsequent T cell immune response can result in either an allergenic Th2/eosinophilic IgE-mediated inflammation or an inflammatory Th1/neutrophilic cell influx into the airway. The Th2 allergenic response involves the interaction of DCs, Th2 cells, and IL-4 producing basophils which induce the expansion of type 2 innate lymphoid cells (ILC2) which also produce the Th2 cytokines, IL-5, IL-9, and IL-13, leading to eosinophil and mast cell trafficking to the lung and goblet cell mucus secretion (70) (Figure 4). IL-4 derived from Th2 cells also induces IgE production by B cells. The mixed Th2 and Th1 neutrophilic or Th2 low asthma is induced by toll-like receptor (TLR) activation resulting in IL-1β secretion and activation of inflammatory Th1 and Th17 cells. These cells release IL-17a and IFNγ, which activate neutrophils and macrophages to release TNFα and induce inflammatory signals. The resulting immune infiltrates induces the symptoms of asthma—bronchoconstriction, mucus production, and the resultant tissue remodeling increasing smooth muscle and collagen deposition (71, 72). The subsequent remodeling results in airway wall thickening, compromised lung function and changes in the lung microbiota (41, 73). The mechanisms of asthma have been studied successfully over the decades using murine models, such as the ovalbumin and the house dust mite challenge models (74). While all models have limitations, these studies have led to successful therapy development and understanding of the genetics of asthma (75). Asthma treatment targets immune processes by using inhaled corticosteroids, to reduce inflammation, and bronchodilators to counteract the effects of bronchoconstriction induced by the release of histamine, prostaglandins, interleukins, or leukotrienes.

Several genetic studies have identified asthma susceptibility genes, including IRAK3, SMAD3, ORMDL3, IL-1RL1, IL-13, IL-33, TNFAIP3, and TSLP (76) (Figure 1). Recent studies have highlighted the role of the site of the mutation and allele frequency and the particular site of functionality, such as the asthmatic epithelium, and the epigenetic regulation thereof as being key factors contributing to asthma (77, 78). Studies on DNA methylation, and microRNA modulation of gene expression, are now shedding light on the pathogenesis of this multifactorial disease.

There is increasing evidence that the gut plays a key role in effecting the allergic immune response. Murine models have demonstrated how feeding of gut commensals can reduce allergy symptoms by inducing T regulatory cells (Tregs) which migrate to the lung and reduce the immune response (79, 80). Indeed, antibiotic-mediated disruption of the gut microbiota and mycobiota has been shown to exacerbate allergic asthma symptoms in mice (81, 82). Recently, an elegant study by Arrieta and colleagues found that the relative abundance of the bacterial genera *Faecalibacterium, Lachnospiria, Veillonella*, and *Rothia* and *Clostridium neonatale* are decreased in the gut of children at risk of asthma development (83, 84). These microbes were significantly different between the groups at 3 months of age and the difference decreased as children reached 1 year of age highlighting a colonization window of opportunity and of an appropriate immune education.

## Regulation of Microbiota by Diet

Diet has a major impact on human health, whether by affecting the host directly or through changes of the microbial community. The microbial community in the mammalian gut is a complex and dynamic system with a steady state (85), which can be perturbed by many environmental factors, including diet, lifestyle, drugs, thereby changing the host’s physiology (10, 86) (Figure 2). As a response to dietary changes, shifts in the composition of the gut microbiota occur. Especially during early-life events like weaning off and introduction to solid food, the dynamics of colonization are critically involved in educating as well as training the immune system (87). The consumption of a Westernized diet containing excessive amounts of refined and processed foods, red meats, and sugary beverages, accompanied with a low consumption of fibers, fruits, and vegetables, is associated with the increased occurrence of metabolic diseases, such as diabetes and obesity, both of which are associated with systemic low-grade inflammation attributed to endotoxemia (88, 89) (Figure 2B). A healthy gut microbiota maintains a symbiotic relationship within the gut mucosa offering essential functions in metabolism, immunology, and protection of the host (Figure 2A). The commensal microbiota, consisting of up to 1 × 10^14^ bacteria, confers colonization resistance against pathogens, which is a key host defense mechanism against enteric infections. Commensal microbes occupy niches and exhaust nutrients thereby limiting the growth of newcomers. Dietary changes, inflammation, and antibiotics can disrupt the commensal microbial community and hence increase the risk of colonization and expansion of incoming pathogens (90). The commensal microbiota is also essential for the elimination of pathogens from the gut. For example, unlike conventional specific pathogen free mice, GF mice are unable to eradicate enteric pathogens such as *Citrobacter rodentium* from the gut (19). Therefore, the abundance and diversity of microbial members plays a crucial role in fulfilling these functions, i.e., symbiosis, colonization resistance, clearance of pathogens, etc. Decreased microbial diversity or altered composition, e.g., increased *Firmicutes* to *Bacteroidetes* ratio, entail various health risks for the host and are generally associated to poor health (91). Feeding high-fat and high sucrose diet to wild-type (WT) mice leads to decreased gut microbiota diversity and an increase in opportunistic pathogens, resulting in a decreased prevalence of specific gut barrier-protective bacteria (92). However, reverting the animals to regular chow food reverses the dietary perturbations thereby confirming the structural resilience of the gut microbiota. Similarly, the human microbiota can rapidly adapt to dietary changes (93, 94). A comparative analysis of vegan, vegetarian, and omnivore diets and the corresponding shifts in microbial communities revealed that a significant increase in β-diversity was present as quickly as 24 h post switch to the animal-based diet (93). These findings suggest that changes in environmental conditions of gut microbiota, e.g., through changes in diet, put selective pressure on various species, which in turn leads to competition for the most adaptable bacteria to survive and replicate (7).

## Diet and Immune Responses in the Adipose Tissue

The adipose tissue is an active endocrine and secondary immune organ consisting of adipocytes, immune cells (T cells and macrophages) and connective/nerve tissue which produces hormones including adipokines [such as leptin and resistin (pro-inflammatory) and adiponectin (anti-inflammatory)], cytokines, and chemokines. The adipose tissue in lean individuals is characterized by an anti-inflammatory cytokine and adipokine profile (e.g., IL-4, IL-10, IL-33, adiponectin) produced by M2 macrophages and Tregs, while obese mice present an initial CD8^+^ T cell infiltration followed by macrophages resulting in a pro-inflammatory (Th1/Th17 and M1) profile. M1 macrophages secrete pro-inflammatory cytokines, including TNFα, IL-1β, and IL-6 (95). Although a correlation between obesity and IBD is not confirmed [reviewed in Ref. (96)], the cytokine profile of the adipose tissue in patients with IBD and especially in CD patients, is similar to obese individuals exhibiting increased levels of TNFα, IL-6 and leptin and a reduction in adiponectin (97–100). CD adipocytes express TLRs, display a higher presence of commensal bacteria (*Enterococcus faecalis*) and an increased translocation of intestinal bacteria resulting in an increased C-reactive protein production (101). These findings indicate that adipocytes participate in the antimicrobial response and represent a barrier to maintain homeostasis and link the adipose tissue with innate immune responses (102). A typical feature of patients with CD is an enlarged mesenteric tissue wrapped around the intestine, so-called “creeping fat.” This fat is usually found adjacent to inflammatory lesions, it correlates with disease activity, is characterized by high infiltration of lymphocytes and macrophages, high levels of peroxisome proliferator-activated receptor γ (PPARγ) and TNFα and fibrosis (103–105). PPARγ is a nuclear receptor that controls the expression of a large number of regulatory genes in lipid metabolism, insulin sensitization, inflammation, and cell proliferation (106, 107) and can inhibit the activation of nuclear factor κB (NFκB), mitogen-activated protein kinase (MAPK), and cyclooxygenase 2 (COX-2) pathways leading to reduction of pro-inflammatory mediators (cytokines and prostaglandins). These findings indicate that mesenteric obesity may play an important role in CD pathogenesis. Contrary to IBD, an association between obesity and asthma, with increased asthma disease severity, has been specifically identified in children. Obesity appears to increase injury in the lungs of asthmatic patients by increasing eosinophil numbers to the airway wall and the systemic production of pro-inflammatory cytokines TNFα, IL-6, IL-1 (5). A recent publication highlighted a pathogenic role for IL-17a produced from ILC3 cells in airways disease in mice with diet-induced obesity. This was accompanied by NLRP3 inflammasome and IL-17a activation in the adipose tissue and the lungs, leading to the identification of a new inflammasome/Th17 mechanism in asthma (108). Similarly to IBD, a reduction in adiponectin levels has also been reported, which can affect the anti-inflammatory immune function in asthma patients (5).

## Dietary Patterns in IBD and Asthma

Dietary nutrients shape the intestinal environment by having a crucial impact on intestinal microbial populations and immune responses. To date, epidemiological evidence from observational studies indicate that intake of fiber rich food, such as fruits and vegetables, can protect against IBD and asthma. Conversely, this protective effect has not been confirmed in randomized controlled trials. Recently, the evidence for the airway and gut microbiota in effecting asthma development and induction is mounting. Timing of neonatal exposure to microbes and the diversity of the exposing environment and gut metabolites appear to effect asthma development (83). In a seminal work by Gevers et al., microbial alterations in naïve treated pediatric CD patients were correlated with certain microbes, specific location and effect of antibiotic treatment—findings that can pave the way for new CD diagnostic tools (109). The effect of dietary habits on the early development of these diseases is yet to be discovered. In the next section, we summarize studies covering different dietary patterns and their contribution to disease status. For a more comprehensive review on dietary advice and interventions see recent reviews (27, 110–112).

### Fat and Sucrose

A recent report from the European Prospective Investigation in Cancer (EPIC) study, did not identify a correlation between body mass index (a measure of obesity) and IBD morbidity (113), therefore proposing that a hypercaloric diet *per se* is not enough to trigger the development of IBD. Epidemiological studies indicate an increased risk of IBD is associated with a higher consumption of red and/or processed meat, dietary fat [especially n-6 polyunsaturated fatty acids (PUFAs)] and low levels of vitamin D (VitD) (4, 114, 115). In addition, an association between disease activity and intake of total fat [trans, saturated, and monounsaturated fatty acids (MUFAs)] and a high n-6/n-3 PUFA ratio has been identified in patients with CD (116). Also, a direct correlation of colonic cytokine levels with saturated fatty acids (SFA) was identified in patients with UC (115). Similarly to IBD, the quality of fatty acids (FAs) appear also to effect the asthma response as trans FAs margarine (a trans FA source), n-6 FAs intake, and oleic acid intake are associated with increased asthma risk (117, 118). In addition to saturated fats, a higher prevalence and preference for foods with high calories, i.e., from fat and sucrose, has been linked to the worldwide increase in metabolic diseases (119). In IBD, one of the first studies that related sucrose to the development of CD was published four decades ago (120). More recently, the EPIC study uncovered an association between high consumption of sugar and soft drinks accompanied by a low vegetable intake to a higher UC risk (121). Similarly, high sugar consumption in sweetened beverages has been linked to asthma and dental caries which is also increased in asthmatics (18, 21, 122). Studies in experimental models, e.g., transgenic CEABAC10 mice fed a diet high in fat and sucrose, resulted in altered microbiota composition, particularly in the expansion of and colonization of AIEC and reduction in protective bacteria, increased permeability and colonic pro-inflammatory mediators (Figure 4), and reduction in short-chain fatty acids (SCFA) concentrations and the SCFA receptor GPR43, supporting a role of fat and sucrose in exacerbating inflammation due to changes in microbial composition (20, 123). Overall, the studies to date indicate that a diet rich in saturated fat and high sucrose content presents a risk factor for IBD and asthma and is associated to inflammation while results from MUFA-containing diets are contradictory.

### Dairy Products

Dairy products are a major source of SFA present in our diets. In a Japanese study, an increased incidence of CD was strongly correlated to milk protein (124). Furthermore, an increase in cheese consumption is associated with an increased risk of both UC and CD (125). In line with these findings, IL-10^−/−^ mice fed a saturated milk fat-derived diet resulted in an increased severity of colitis associated with a colonic Th1 profile and presence of CD4^+^ IFN-γ^+^ cells in the mesenteric lymph nodes (MLNs) as a result of blooming of an opportunistic bacteria *Bilophila wadsworthia* (126) (Figure 4). Interestingly, WT mice fed milk fat diet and presenting a blooming of *B. wadsworthia* did not develop colitis, indicating the impact of genetic predisposition on the subsequent inflammatory response. Of note, 20% of patients with UC benefited from excluding milk and cheese from their diet (127). These collected data indicate that dairy products may play a role in IBD pathology. In contrast to IBD, a decreased asthma risk is associated to milk fat (117, 118). Additionally, it has been reported children who consumed raw milk during childhood show a reduced risk of developing atopy and/or asthma (128–130). The protective effect of raw milk has been speculated to be due to improvement in nutrition, prevention of lactose intolerance, or the presence of “good” bacteria. However, the topic is still debatable and more studies are needed.

### Emulsifiers

Processed foods have been identified as a risk factor for IBD (4, 115) and in 2013, it was hypothesized that the increased incidence in CD was the result of a higher consumption of emulsifiers in processed foods (131). Indeed, using an animal model of colitis (IL-10^−/−^ mice), it was shown that exposure to two common emulsifiers, carboxymethylcellulose (CMC) and polysorbate-80 (P80), aggravated colitis by increasing gut permeability, reducing mucus thickness, promoting higher penetration of intestinal bacteria, and altered microbial composition, particularly by enrichment in *Bilophila* spp. (Figure 4). Exacerbation in obesity/metabolic syndrome was also observed in emulsifier-treated TLR5^−/−^ mice (132). Supporting these findings, it was shown *in vitro* that addition of P80 induced a higher translocation of *E. coli* across M-cells (133) (Figure 4). More recently, using an *ex vivo* model of human microbiota culture which was exposed to CMC and P80 revealed an altered gene expression and microbial composition by, e.g., induction of bioactive flagellin. However, transfer of emulsifier-treated microbiota to GF mice resulted in a low-grade inflammation and metabolic syndrome features but not colitis (134). The relevance of these findings for the development of the microbiota and immune system and in other chronic conditions is yet to be addressed.

### Fibers, Vegetables, Fruits, and Fish

In contrast to dietary fats, diets rich in fish (n-3 PUFAs), fermentable fibers and vegetables and fruits lower the risk for IBD (4, 115). For example, an inverse correlation of eicosapentaenoic acid (EPA) and docosapentaenoic acid with colonic cytokine levels was identified in UC patients (115). Several studies have suggested that a diet low in refined carbohydrates could be beneficial in the treatment of CD, reviewed in Ref. (135); however, clinical trials are needed for this view to be proven (136, 137). Findings from a prospective study investigating the long-term intake of dietary fiber and risk of incidence of CD and UC in women revealed a 40% reduction in risk of developing CD while no association to UC was observed (138). Much of the fiber intake in this study originated from fruits. The Aryl hydrocarbon receptor (AhR) was identified as a potential mechanism linking the positive effects of fruit-derived fiber. AhR is expressed ubiquitously in vertebrate cells and mediates the toxicity of xenobiotic molecules by binding to the AhR nuclear translocator and activating dioxin- or xenobiotic-response element sequences (139). Indole-3-carbinol, a major component of cruciferous vegetables (e.g., broccoli, cabbage, and cauliflower) can activate AhR in the intestine (140), inducing maintenance and expansion of intestinal intraepithelial lymphocytes (IELs) and IL-22-producing ILCs (141). IELs are T cells expressing αE (CD103)/β7 integrin, localized in between epithelial cells and are characterized as either conventional (CD4^+^ or CD8αβ^+^TCRαβ^+^) or unconventional (TCRγδ^+^ and CD8αα^+^TCRαβ^+^) IELs. IELs have been regarded as a first line of defense against infected/damaged epithelial cells and are implicated in the regeneration of the intestinal epithelium (142). Indeed, recent studies have demonstrated that IELs play a hitherto underappreciated role in gut epithelial homeostasis (143). In addition, aberrant IEL phenotype and lineages are now evident in pathologies such as celiac disease and enteropathy-associated T lymphoma (144). The roles that these differing IELs play in sensing and interacting with the gut microbiota in IBD and other GI conditions warrants further investigation.

In asthma, a high-fiber intake in late pregnant mothers was correlated with high serum acetate levels and resulted in lower infant GP visits for cough or wheeze (17). In children aged 10–14, a dietary intake of fruits, but not vegetables, was negatively related to wheeze, while no protective effect was identified in adults (27).

Intake of oily fish, such as salmon, sardines, herring, tuna, and mackerel, which are rich in n-3 FAs, have shown a potential benefit in preventing asthma in children and in patients with UC, while no beneficial effect in adults with asthma was reported (145, 146).

## Nutrients and Their Impact on Microbiota and Immune Responses at Mucosal Sites

Recent evidence has identified the existence of a cross talk between the host and the commensal microbiota within the gut. In this dialog, nutrients play an important role either by directly interacting with the host *via* the epithelium or the intestinal immune system or indirectly, by modulating the composition of the commensal microbiota which in turn will interact with the immune system, and *vice versa* (Figure 2A). The immune system will react promptly and adapt depending on the microbiota (commensal, pathobionts, and pathogens) and the diet (prebiotics, supplements, or detrimental nutrients). The worldwide increased incidence of IBD and asthma has been hypothesized to be associated with changes in dietary habits, i.e., westernized life style. In the following sections, we will outline several important macro- and micronutrients associated with diets and their effect on immune responses at mucosal sites important in health and in IBD and asthma.

### Macronutrients

In this section, we will summarize the impact of the main macronutrients fat, carbohydrates and proteins on immune responses and microbiota (Table 1).

#### Fats

Under this section, we will outline the impact that saturated-, monounsaturated-, and PUFAs have on immune and microbial responses associated with IBD and asthma.

##### Saturated Fatty Acids

Fatty acids belonging to SFAs and containing 12 or less carbon (C*X*) atoms, include carprylic acid (C8:0), capric acid (C10:0), and lauric acid (C12:0), are found in vegetable oils, cocoa butter, palm oil. SFAs containing more than 12 carbon atoms include myristic (C14:0), palmitic acid (C16:0), stearic acid (C18:0) which can be found in lard, butter, beef, pork, chicken fats, eggs, and vegetable oils (147).

Evidence exists that SFA can act as pro-inflammatory mediators, e.g., as ligands for TLR4 (148, 149). Potential mechanisms by which SFAs elicit a TLR4-induced inflammatory response have been recently reviewed (12). Briefly, it is proposed that similar to lipopolysaccharide (LPS), the SFA lauric acid can trigger TLR4 *via* CD14/MD2 activation thereby promoting the expression of the transcription factor NF-κB, which plays a crucial role in the induction of the pro-inflammatory mediators COX2, TNFα, IL-1β, IL-6, CXCL8, IL-12, and IFNγ (150) (Figure 4). Comparably, palmitic acid and stearate can induce pro-inflammatory cytokine production from macrophages *via* degranulation of Ikappa B alpha (IκBα) and phosphorylation of c-Jun N-terminal kinases, MAPKs, and extracellular signal-regulated kinases (ERK). LPS together with palmitate activate reactive oxygen species and the NLRP3 inflammasome leading to IL-1β maturation in macrophages (95) (Figure 4). Moreover, a high intake of SFA, i.e., given in western diet, leads to the modification of the gut microbiota raising the proportion of Gram-negative bacteria and thereby the natural ligand for TLR4, LPS, as well as an increase in intestinal permeability, which in itself induces a state of metabolic endotoxemia (151) (Figure 4). Intake of SFAs can also increase plasma low-density lipoprotein cholesterol by inducing the formation of low-density Lipoprotein (LDL) and by reducing LDL turn-over leading to the generation of oxidized LDL (152). Both oxidized LDL and phospholipids are damage-associated molecular patterns, which are also recognized by TLR4 and can trigger a CD36–TLR4–TLR6-mediated inflammatory response (153). These findings support a pro-inflammatory effect of SFA on the microbiota on innate responses in macrophages.

To date, mechanistic studies on saturated fats in human IBD are scarce and, therefore, much of our knowledge on SFAs and intestinal inflammation emanates from studies in experimental models. TNFΔARE mice fed a palm oil-based high-fat diet for up to 12 weeks resulted in an initial acceleration of ileitis followed by worsening of proximal colitis associated with loss of TJ protein occludin in the distal ileum, endotoxin translocation, and increased infiltration of DCs and Th17 cells into the lamina propria but without the development of obesity or obesity-associated metabolic features (154). Similarly, Mdr1a^−/−^ mice fed a lard-based high-fat diet for 12 weeks led to an exacerbation of spontaneous colitis associated with elongated crypts, loss of goblet cells, and infiltration of immune cells. Contrary to TNFΔARE mice, Mdr1a^−/−^ mice developed obesity as characterized by increased adiposity and presence of foamy macrophages, while WT mice did not (155). Rats fed a diet containing capric and lauric acid followed by DSS-induced colitis, developed worse colitis associated with a higher colonic myeloperoxidase activity and a pro-inflammatory cytokine profile as well as a reduction in goblet cells (156). Overall, these findings indicate that the type of SFA diet, microbiota status, diet regimen, and/or the genetic background of the animals determine the development of intestinal inflammation and obesity, suggesting that different dietary-induced mechanisms regulate these two conditions. In asthma, SFA have been shown to effect symptoms and immune activation, e.g., by inducing a neutrophilic inflammation and suppressing bronchodilator recovery in asthmatic patients (11, 27) (Figure 4).

##### Monounsaturated Fatty Acids and Derived Oils

Monounsaturated fatty acids, including palmitoleic acid (C16:1) and oleic acid (C18:1, OA) are normally found in macadamia nuts, blue-green algae, olive oil, canola oil, beef tallow, lard, and avocado.

Diets rich in MUFA appear to reduce LDL cholesterol and potentially increase high-density lipoprotein (HDL) cholesterol (157), and palmitoleate treatment of M1 macrophages induce an anti-inflammatory M2 profile (158) indicating an anti-inflammatory capacity of MUFAs. The specific role of MUFAs in IBD and asthma remains inconclusive. For example, a prospective study by de Silva and colleagues showed that a dietary oleic acid was inversely associated with UC development (114), while palmitoleic and oleic acid treatment of polarized intestinal epithelial cells impaired epithelial barrier function (159). MUFA and oleic acid intake indicated an increased risk of wheeze and non-atopic asthma, respectively (160). Extra virgin olive oil, high in MUFAs, contains highly bioactive components which are present in the unsaponifiable fraction (UF). Beneficial effects of UF and olive oil were demonstrated in an acute DSS model of colitis and in mice with *C. rodentium* induced colitis. Disease amelioration included alleviation of oxidative stress, reduction of pro-inflammatory proteins and increased levels of intestinal alkaline phosphatase, which can de-phosphorylate bacterial LPS (161–163). In line with these findings, isolated blood and intestinal T cells from UC patients treated with UF resulted in a reduction in T cell activation, β7 integrin expression and IFNγ production as well as induction of apoptosis (164). Findings from these studies indicate MUFA exert both pro- and anti-inflammatory activities in these mucosal conditions.

##### Polyunsaturated Fatty Acids

Polyunsaturated fatty acids are FAs containing more than one double carbon bonds. Therefore, naturally, they are more prone to oxidation and oxidized LDL synthesis. Long chain PUFAs are divided in two main groups; omega-3 (n-3) PUFAs including—alpha-linolenic acid (ALA, C18:2), docosahexaenoic acid (DHA, C22:6), and EPA (C20:5); and omega-6 (n-6) PUFAs including—linoleic acid (LA, C18:3), and arachidonic acid (ARA, 20:4). LA and ALA are referred to as essential FAs as they are the precursors of ARA, EPA, and DHA. DHA and EPA compete for the enzymes and products of ARA metabolism whereby they can antagonize the formation of inflammation related eicosanoid mediators (165, 166).

Arachidonic acid is the primary n-6 PUFA found in inflammatory cells and is important for the production of inflammatory eicosanoids. ARA is formed out of LA which is further converted to prostaglandins [e.g., prostaglandin E_2_ (PGE_2_), leukotrienes (LTBD_4_), and other lipoxygenase or cyclooxygenase products (COX1/-2)], the so-called eicosanoids, all of which have pro- and anti-inflammatory in addition to atherogenic and pro-thrombotic effects (166). PGE_2_ is one of the key prostaglandins produced in the intestine where it has dual functions: (1) a pro-inflammatory role, e.g., it is produced by macrophages and neutrophils as a response to inflammatory stimuli and (2) a regulatory role, by inducing immune tolerance, independent of IL-10 or Tregs (167).

n-3 PUFAs are primarily sourced from the human diet, with DHA and EPA especially sourced from fish (e.g., salmon) and ALA from seed oils (e.g., walnut, linseed oil). Several reports have highlighted their effect in preventing and/or treatment of different inflammatory diseases in animals and humans, including IBD and asthma. n-3 PUFAs can inhibit TLR4 signaling and the subsequent gene transcription of pro-inflammatory mediators (168), a process partly mediated *via* GPR120 (169) (Figure 3). n-3 PUFAs can also activate the anti-inflammatory transcription factor PPAR-γ and inhibit NF-κB (Figure 3) and the subsequent pro-inflammatory cytokine production including TNFα, activity which is highly expressed in the mucosa of patients with IBD and asthma subtypes (13, 170, 171). DHA can also improve epithelial barrier integrity by increasing the expression of the TJ proteins occludin and claudin-1 (Figure 3) (172), as the integrity of the epithelium is critical in preventing paracellular translocation of LPS into systemic circulation. PUFA supplementation in particular those related to fish oils can also modulate asthma symptoms (173, 174), with reduction of wheeze, improved pulmonary function and reduced pro-inflammatory mediators in sputum. However, it should be noted that many trials are designed to measure different outcomes which can deliver conflicting findings (27, 175). With the evolution in food technology and modern agriculture in the last 100 years the amount of n-6 FAs, e.g., LA present in our food has increased due to change in animal feed from grass to grains (176, 177). Therefore, the ratio of n-6/n-3 PUFAs present in the food has changed from 1:1 to 16:1 in USA/Europe (177) (Figure 2). This ratio has increased dramatically due to food processing, less fish and fiber consumption, and the dietary habits of farm animals. In line with this, a recent study identified a higher ARA:EPA ratio in the inflamed mucosa of UC patients, which correlated with the severity of the disease (178). No association of n-6/n-3 ratio was found in individuals with hay fever or allergic sensitization (179). The relevance of a lack of n-6/n-3 ratio association to asthma is still to be uncovered.

#### Carbohydrates

Carbohydrates are divided into four groups: monosaccharides, disaccharides, oligosaccharides and polysaccharides. Generally, monosaccharides and disaccharides are referred to as sugar. In the western diet a large amount of calories are ingested in form of refined carbohydrates, i.e., sucrose, starch, fructose syrup, etc.—obtained from soft drinks, pastries and desserts, and white bread. This energy-dense but nutrient-poor diet is a risk factor for obesity, type 2 diabetes, cardiovascular diseases and more. Therefore, the biological plausibility exists that it has impact on intestinal inflammation and asthma. Moreover, related to the carbohydrates that can be metabolically used by gut microbes, the term “microbiota-accessible carbohydrate” has been proposed. The term refers to the ability of microbial carbohydrates to modify the composition of the microbiota, and dictate the functionality and metabolic output (180).

##### Fibers

Dietary fiber is a plant-based nutrient and a type of carbohydrate, which due to its biochemical structures resists digestion by intestinal and pancreatic enzymes in the human GI tract. Therefore, the fiber passes through the GI tract relatively intact. Fermentable carbohydrate substrates such as non-starch polysaccharides, resistant starch and oligosaccharides serve as important substrates for the gut microbiota. The microbes located in the human colon use fermentation to produce SCFAs, lactate and gas (181). These fermentation products selectively promote the growth of beneficial Bifidobacteria and Lactobacilli and exert anti-inflammatory (inhibition of NFκB transcription *via* GPR41) and anti-carcinogenic functions (182).

###### Short-Chain Fatty Acids

Upon fermentation of dietary fiber, bacterial metabolites such as SCFAs are produced in the colon. SCFA mediates the communication between the commensal microbiota and the immune system affecting the balance between pro- and anti-inflammatory responses. The beneficial effects of butyrate on colonic health are particularly well established (183). Microbial-derived butyrate and to a lesser extent acetate and propionate, can facilitate the generation of extrathymic Foxp3^+^ Tregs which are crucial for limiting intestinal inflammation (17, 184, 185) (Figure 3). Presence of butyrate during human DC maturation results in a tolerogenic phenotype, with an increased expression of IL-10 (186, 187), further supporting the regulatory potential of SCFAs (Figure 3). SCFA can signal through the activation of specific G-protein coupled receptors (GPCRs), GPR41, GPR43, GPR109, which are particularly expressed in immune cells, e.g., polymorphonuclear leukocytes, and are suggested to participate in immune surveillance of the colonic mucosa affecting the balance between pro- and anti-inflammatory responses (188). GPR43 is additionally expressed in the colonic epithelium where it can mediate SCFA-regulated effects on epithelial barrier and proliferation (Figure 3). Butyrate is also a natural ligand for PPARγ, which is expressed in colonic epithelial cells, macrophages and lymphocytes. Colonic PPARγ expression is linked to host–microbe interactions with natural (e.g., SCFA—butyrate, conjugated LA) and synthetic (e.g., 5-aminosalicylic acid) PPARγ ligands preventing inflammation in experimental colitis (189).

A significant decrease in the number of butyrate-producing bacteria including *Eubacterium rectale/Roseburia* spp (which belong to *Clostridium coccoides*) and *F. prausnitzii* (which belong to *Clostridium leptum* cluster), both within the Firmicutes phylum was revealed in patients with UC and CD (190, 191). In patients with UC, colonic irrigation with butyrate is able to limit inflammation and in experimental models it ameliorates inflammation and modifies microbial composition (183, 192).

In the Canadian CHILD study the SCFA acetate was reduced in the feces of infants with atopy and wheeze (83). Feeding a high fiber diet to mice has resulted in gut microbiota alteration which concomitantly leads to increased serum SCFAs, such as acetate and propionate, and alleviates allergic asthma symptoms (Figure 3). These SCFAs induced an enhanced DC and macrophage phagocytosis and reduced Th2 responses in the murine lung (17, 23, 193). Furthermore, binding of propionate to GPR41 and acetate and propionate to GPR43 also supported the reduction in airway inflammation (17, 23, 194, 195). These microbiota-mediated effects are also linked to maternal influences of asthma risk, where high serum acetate concentrations, but not propionate, in pregnant mothers’ correlate with reduced asthma risk and are thought to modulate Tregs biology of the fetus (17). These findings clearly point to a crosstalk between the gut and the lung *via* SCFA in asthma and between the microbiota and the intestine in IBD, indicating that treatments aiming to increase SCFAs and SCFA producing bacteria should be further investigated.

#### Proteins

Proteins consist of carbon, hydrogen, oxygen, and nitrogen elements. They are essential nutrients and are involved in virtually all physiological functions. High protein intake, especially animal derived protein, is associated with an increased risk of CD (196). In asthmatics high ingestion of cured meats is linked with worsening of symptoms (24).

Carnitine is an amino acid derivative synthesized primarily in the liver and kidneys from lysine and methionine and is involved in lipid metabolism in eukaryotic cells (197). In humans, the main source of carnitine is red meat. Humans with an omnivorous diet following ingestion of l-carnitine, presented increased plasma trimethylamine-N-oxide (TMAO) levels, which was dependent on microbiota mechanisms, when compared to vegans or vegetarians. Elevated plasma levels of TMAO are positively correlated with an increased risk for major adverse cardiovascular events (198). However, TMAO has also protective functions, e.g., by protecting cells from osmotic and hydrostatic damage, and therefore, is essential for all organisms (25). A recent study proposed plasma TMAO as a non-invasive biomarker for IBD, as decreased TMAO levels are seen in these patients (199). The reduced TMAO was likely related to alterations in the gut microbiome (the abundance of anaerobes or facultative anaerobes) in these patients. TMAO is also an oxidation product of trimethylamine which can be found in seafood, fish, etc. Consumption of TMAO-containing food, e.g., oily fish can lead to accumulation of TMAO and protection against asthma in childhood (200). Further studies are necessary to shed a light into mechanisms that underlie the association of high intake of animal protein in these conditions.

Other studies have also shown how dietary peptides and amino acids can modulate intestinal immune functions and influence inflammatory responses. Supplementation with the dipeptide alanine-glutamine, led to decreased expression of inflammatory mediators and increased expression of mucin 2 (MUC2) promoting mucosal recovery in the DSS-induced colitis mouse model (26). Moreover, lower serum tryptophan is associated to active CD (201). Supplementing tryptophan presented beneficial effects in the DSS-induced porcine IBD model, by inducing T cell apoptosis and thereby inhibiting Th1-mediated immune responses and subsequently reducing inflammation (202).

### Micronutrients

Micronutrients or trace elements are nutrients required by organisms in small quantities to maintain a variety of physiological functions and as most of them are essential, they need to be obtained from the diet. These minerals include iron, cobalt, chromium, copper, iodine, manganese, selenium (Se), zinc (Zn) and vitamins include Vitamin A (VitA), vitamin B1 (VitB1), Vitamin B6 (VitB6), Vitamin B9 (VitB9), VitB12, VitD, and VitK. Micronutrient deficiencies impair immune function and increase the severity of disease (203). Micronutrient deficiencies occur in more than half of patients with IBD, with CD patients presenting more deficiencies than UC patients, with the most common being VitB1, VitB6, VitB12, VitD, VitK, iron, folic acid, Se, and Zn (204). This deficiency in vitamins (hypovitaminosis) is thought to be the result of malabsorption, altered microbial composition and impaired host mucosal system. Low dietary intakes of VitA and VitC are associated with asthmatics (205). The next section summarizes the most relevant micronutrients in relation to IBD and asthma pathology.

#### Vitamins

Vitamins can be absorbed from the diet but the gut commensal microbiota play an important role in their production and bioavailability (206). Indeed, the diet of germ-free mice requires supplementation with dietary VitK and B Vitamins to maintain a normal function (207).

##### Vitamin A

Vitamin A is a group of unsaturated organic compounds including retinol, retinoic acid (RA), and several pro-vitamin A carotenoids including beta-carotene. They are fat-soluble substances obtained from animal food sources. RA is a metabolite of VitA, which is produced by CD103^+^ DCs and epithelial cells and acts as ligand for RA receptors (RARs) and Retinoic-X-Receptor (RXR), transcription factors regulating gene expression. Lymphoid cells express RAR, RXRs and RA, which are known to regulate IgA and mucosal homeostasis (Figure 3). DCs from Gut associated lymphoid tissue (GALT) produce RA to sustain gut tropism and in synergy with GALT-DC-derived IL-6 or IL-5, induce IgA production (208) (Figure 3). RA can also control the presence of RORγ^+^ ILCs, the formation of lymphoid tissue in the small intestine (209) and appears to be a cofactor in IgA class switch recombination (210). RA can also induce the gut-homing capacity on T cells by the up-regulation of the integrin α4β7 and the chemokine receptor CCR9 (211) (Figure 3). A diet deficient in VitA can lead to a systemic pro-inflammatory state, due to a lack of homing integrins in MLN activated T- and B-cells, which then go into systemic circulation instead of migrating back to the gut (212, 213). RA, together with TGFβ, promotes naïve CD4^+^ to become Foxp3^+^ Tregs and RA alone has also inhibitory effects on Th17 cell differentiation (Figure 3). Others have also described that VitA can impair the reprogramming of Tregs into IL-17-producing cells during intestinal inflammation (214).

Patients with IBD have been reported to be deficient in VitA (Figure 4). Cytochrome P450 26 B1 (CYP26B1) participates in the degradation of RA, and homozygous carriers of the CYP26B1 polymorphism rs2241057 have been associated as risk factor of CD development, linking an elevated catabolic function of RA to IBD (215). Supplementation of VitA/RA seems to attenuate intestinal inflammation in experimental models and even induces a shift in Th17/Tregs in UC biopsies (216–218). VitA appears to influence the microbiota, as its deficiency seems to favor a non-symptomatic reservoir of *E. coli*-like enteric infections (219, 220).

##### Vitamin D

Vitamin D belongs to a group of fat-soluble vitamins which are essential for bone mineralization and optimal intestinal absorption of calcium, iron, magnesium, phosphate, and Zn. VitD regulates the epithelial integrity/barrier function and is involved in the detoxification and protection against infection as well as in controlling of the commensal microbiota (221, 222).

Vitamin D can be obtained from the diet or by dermal synthesis, e.g., in the skin where it is produced from 7-dehydrocholesterol (222). 1,25-dihydroxyvitamin D3 [1,25(OH)2D3] is the active form, which arises from the bloodstream (endocrine action) or it can be locally produced from circulating 25(OH)D3 within intestinal cells (intracrine, autocrine and paracrine action). VitD is ubiquitously expressed in several human tissues including immune cells, but its expression is higher in intestinal epithelial cells (223). VitD deficiency has been associated with a greater disease activity and extended disease duration in patients with IBD (Figure 4) – why a supplementation of VitD is often required. Human polymorphisms in the vitamin D receptor (VDR) are also associated with susceptibility to IBD (224). Vitamin D3 has been linked to beneficial effects in asthma; however, the benefits are mainly observed in children or *via* maternal supplementation (225). Recent studies have identified that VitD3 appear to modify VEGF function and reduction in airway smooth muscle proliferation (226).

The biological actions of 1,25(OH)2D3 are mediated *via* the VDR, which acts as an heterodimer with RXR to activate VitD target genes (222, 227). VDR targeted pathways regulating inflammatory responses include TLR and NF-κB signaling, Th17/Tregs response, apoptosis, cell proliferation and differentiation, barrier function, etc. (Figure 3). The anti-inflammatory role of 1,25(OH)2D3 is based on the suppressive effect of NFκB activity, as NFκB-induced pathways are enhanced in VDR^−/−^ mice exposed to bacterial and chemically induced colitis (228). 1,25(OH)2D3 regulates intestinal barrier trough the up-regulation of TJ proteins including occludin, ZO1, claudin 2 and E-cadherin (221, 229) (Figure 3). In support of this, IL-10^−/−^ mice expressing the human VDR in intestinal epithelial cells resulted in a reduced development of spontaneous colitis (230). VitD has been linked to the modulation and control of the gut commensal microbial composition (Figure 3), since VDR^−/−^ mice present an altered microbiota with more abundance on Bacteroidetes and Proteobacteria phyla and less abundance on the Firmicutes phyla (231). Interestingly, analysis of mice treated with 1,25(OH)2D3 revealed an increased *C. rodentium* load in the colon and spleen doubtless due to the suppression of a Th17 response, which is essential for *C. rodentium* clearance (232). In contrast, following infection with *C. rodentium*, a diet deficient in VitD aggravated barrier function, microbiota composition and inflammation (233). Correspondingly, mice fed a high-fat and VitD deficient diet presented increased ileal antimicrobial peptides and *Helicobacter hepaticus* and reductions in TJ proteins, MUC2 expression and abundance of beneficial bacteria such as *Akkermansia muciniphila* developing insulin resistance and fatty liver (234).

##### Other Vitamins

###### Vitamin B1

Vitamin B1, also known as thiamine, is mainly obtained from whole grains, trout, pork, peas and beans. It has an important role in the catabolism of sugars and amino acids (235, 236). Thiamine is a component of the pyruvate dehydrogenase that catalyzes the formation of Acetyl CoA in FA synthesis, a pathway which is altered in IBD (237). Pediatric IBD patients presented alterations in cellular transport of thiamine and FAs synthesis (e.g., bile acids) (237, 238). Altogether, these findings highlight a potential link between the microbiota, glucose, FAs and mucosal alteration leading to intestinal inflammation in which thiamine may have a key role.

###### Vitamin B6

Vitamin B6 is a water-soluble vitamin, also known as pyridoxine, which exists in several forms and can be obtained from fruits, vegetables, grains, fish and meat. The biologically active form of VitB6, Pyridoxal 5′-phosphate (PLP), is involved in the synthesis or metabolism of proteins, lipids and carbohydrates and important in the modulation of immune pathways (236, 239). PLP treatment ameliorated colitis in IL-10^−/−^ mice due to a reduction in colonic TNFα, IL-6, IFNγ, COX-2 and nitric oxide synthase (iNOS) expression and modulation of the chemotactic lipid S1P (240). *Eubacterium rectale*, a dominant non-pathogenic fecal Gram-positive commensal bacterium has been described as one of the important bacterial group synthetizing PLP (241).

###### Vitamin B9

Vitamin B9 is a water-soluble vitamin also known as folic acid or folate, it can be obtained from vegetables and fruits and is important in DNA repair and methylation aiding rapid cell division and growth during, e.g., infancy and pregnancy. Bacteria linked to folate biosynthesis include *Bifidobacteria* and *Lactobacilli* groups (207, 242, 243). A deficiency in VitB9 has been more commonly ascribed to CD, especially in patients with ileal disease, than in UC patients (244, 245). VitB9 deficiency has also been associated with a reduction in colonic Foxp3^+^ Tregs (33). Folate supplementation during pregnancy has been linked to an increased risk of infants developing asthma; however, this is controversial with conflicting studies providing little clear evidence (34, 246).

###### Vitamin K

Vitamin K is a fat-soluble vitamin required for synthesis of certain proteins involved in blood coagulation and is linked to calcium pathways and calcification. VitK1 can be obtained from, e.g., meats, cheeses, and eggs or synthetized (VitK2) by the microbiota of the colon *de novo* or from VitK1 (236, 247, 248). VitK deficiency has been associated with both adult and pediatric CD patients (249). A protective role of VitK was shown in a model of DSS-induced colitis associated with a reduction in IL-6 production from B cells (250).

#### Minerals

Minerals are obtained from the diet, and are essential nutrients needed by organisms for synthesis of common organic molecules. Accordingly, mineral deficiencies in the westernized diet have a major impact on host health. The next section summarizes the most relevant minerals in relation to IBD and asthma pathology.

##### Iron

Anemia is one of the most common extra-intestinal manifestations of IBD. The deficiency, results from either an absolute state, i.e., poor dietary intake of iron, reduced iron absorption, and/or increased blood loss from chronically inflamed intestinal mucosa; and/or functional iron state, i.e., deficiency in VitB12 and insufficient availability of iron for incorporation into erythroid precursors despite normal or increased body iron stores (251, 252). It is estimated that up to 80% of patients with IBD present with anemia (253). Consequently, oral or i.v. iron supplementation is important in treatment of IBD patients. Nonetheless, caution is needed as non-absorbed iron can be toxic to intestinal epithelial cells, since it can stimulate growth and virulence of bacteria and appears to worsen disease activity in the patients (178, 254). In support of this data, rats supplemented with iron and exposed to DSS-induced colitis revealed an increased neutrophil infiltration, TNFα and IL-1 expression and NF-κB activation – all of which could be prevented by supplementing the diet with VitE (dl-alpha-tocopherol acetate) (255). In contrast, rats with humanized gut microbiota and fed dietary iron supplementation exhibited an increased abundance of *Bacteroides* spp. and *Clostridium* cluster IV, leading to an increased butyrate concentration in the gut, without the induction of colitis. Thus, iron supplementation can increase the proportion of beneficial gut microbiota metabolites which may contribute to gut health in IBD individuals (256).

##### Other Minerals

###### Selenium

Selenium is an essential antioxidant trace mineral which can be obtained from proteins or vegetables and is used in the body to synthetize the amino acid selenocysteine (selenoproteins). Large amounts of Se can cause toxicity (257). The amino acid transporters SLC3A1 and SLC1A4 have been suggested as Se transporters (236) and play a role in intestinal epithelial permeability and barrier function. Lower Se serum levels have been described in children with IBD (258). A possible therapeutic role for Se has been described, whereby macrophage derived selenoproteins enhance 15-hydroxyprostaglandine dehydrogenase (15-PGDH) and protects mice from DSS-induced colitis (259). A potential role for Se in asthma pathogenesis has been hypothesized; however, data from human studies are conflicting. A benefit of Se supplementation in animal asthma studies has been identified by regulating Th cell differentiation (260).

###### Zinc

Zinc is an important mineral involved in wound repair, tissue regeneration, and the immune response. Low serum Zn levels have been reported in children with IBD (258). Zn given orally to CD patients restored intestinal permeability by modulating TJ proteins in both the small intestine and colon (261). Zn concentrations have been found to be significantly lower in asthmatic patients and it is thought to result in reduced antioxidant function increasing asthma risk (262).

## Conclusion and Future Perspectives

The increasing incidence of IBD and asthma implies a fundamental role for environmental factors. Several lines of evidence have identified the consumption of a diet high in saturated fat and high in sucrose increases a person’s risk for several chronic conditions including IBD and asthma (4, 27). Studies in animal models, human cells and tissues for both conditions have also highlighted the complex interaction between diet, the microbiota and the host response, especially regarding aggravation of inflammatory responses upon consumption of certain triggering nutrients and diets. Dietary ingredients in the western diet are not limited to the ones listed in this review. Apart from the increased consumption of saturated fats and sucrose, there is an increased consumption of food additives including sweeteners, emulsifiers, thickeners, preservatives and food colorings. Some of these have already been associated to asthma and IBD (122, 132, 134) but there is a need for larger epidemiological studies to identify the effect that these products may have on both microbial composition and host responses (e.g., inflammation, metabolism, etc.) in disease development. In an elegant study by Arrieta and colleagues it was shown that changes in the microbiota especially at a young age, can help to identify a window of opportunity for bacteria colonization and an appropriate immune education in asthmatics (83). Future studies aiming to identify whether a similar new widow also exists prior to IBD onset and whether the education of the mucosal immune system can be regulated by specific diet combinations and corresponding microbial alterations are required. Such studies would likely lead to the discovery of new target/regulatory mechanisms for both conditions.

Individuals with IBD and asthma present with a deficiency in certain essential nutrients, e.g., VitD and Iron. The reductionist approach undertaken whereby the effect of one single nutrient has been investigated in animal models, has so far provided major insights into the regulatory mechanisms associated with inflammation and metabolism. In addition, the majority of animal studies and diets, employs standard “high fat” diet containing a nutrient content designed to sustain rodent health and are, therefore, not relevant to a human and patient dietary intake (263). Studies using more complex systems, e.g., humanized mice (consisting of both a human immune system and human microbiota) fed with a humanized diet will provide a deeper mechanistic understanding of the complex diet-microbiota-host network. Integration of various approaches, e.g., genomic, transcriptomic, metabolomics or systems biology, will reveal a clearer picture of the components contributing to the pathology of these chronic inflammatory conditions. Furthermore, the impact of current therapeutic treatments and diets on disease progression and the microbiota are currently lacking, therefore, future studies addressing this question could lead to new beneficial treatment strategies.

## Author Contributions

DS, MA, and JM wrote the manuscript; SM wrote and edited the manuscript. All authors read and approved the final manuscript.

## Conflict of Interest Statement

The authors declare that the research was conducted in the absence of any commercial or financial relationships that could be construed as a potential conflict of interest.
